# Nanostructured MoS_2_/BiVO_4_ Composites for Energy Storage Applications

**DOI:** 10.1038/srep36294

**Published:** 2016-11-03

**Authors:** Yukti Arora, Amit P. Shah, Shateesh Battu, Carina B. Maliakkal, Santosh Haram, Arnab Bhattacharya, Deepa Khushalani

**Affiliations:** 1 Department of Chemical Sciences, Tata Institute of Fundamental Research, Mumbai-400005, India; 2 Department of Condensed Matter Physics and Materials Science, Tata Institute of Fundamental Research, Mumbai-400005, India; 3 Department of Chemistry, Savitribai Phule Pune University, Ganeshkhind, Pune-411007, India

## Abstract

We report the optimized synthesis and electrochemical characterization of a composite of few-layered nanostructured MoS_2_ along with an electroactive metal oxide BiVO_4_. In comparison to pristine BiVO_4_, and a composite of graphene/BiVO_4_, the MoS_2_/BiVO_4_ nanocomposite provides impressive values of charge storage with longer discharge times and improved cycling stability. Specific capacitance values of 610 Fg^−1^ (170 mAhg^−1^) at 1 Ag^−1^ and 166 Fg^−1^ (46 mAhg^−1^) at 10 Ag^−1^ were obtained for just 2.5 wt% MoS_2_ loaded BiVO_4_. The results suggest that the explicitly synthesized small lateral-dimensioned MoS_2_ particles provide a notable capacitive component that helps augment the specific capacitance. We discuss the optimized synthesis of monoclinic BiVO_4_, and few-layered nanostructured MoS_2_. We report the discharge capacities and cycling performance of the MoS_2_/BiVO_4_ nanocomposite using an aqueous electrolyte. The data obtained shows the MoS_2_/BiVO_4_ nanocomposite to be a promising candidate for supercapacitor energy storage applications.

The ever-increasing global energy demands have spurred increased research into energy harvesting and storage systems^1^,^2^. The development of effective energy storage systems with high energy density as well as high power density is becoming increasingly important. Electrochemical capacitors, also termed as supercapacitors, have attracted significant interest as these devices bridge the energy density gap between conventional capacitors and batteries. Layered inorganic systems exhibit unusual properties that are technologically important. The unique mechanical, electronic, thermal and optical properties of graphene and other two-dimensional layered materials like the transition metal dichalcogenides (TMDCs) such as molybdenum disulfide (MoS_2_) have enabled them to be utilized for various novel applications^3^,^4^,^5^,^6^. One specific application for which these layered materials have been recently explored is in energy storage devices, such as lithium-ion batteries and supercapacitors^7^,^8^. The recent proliferation of research into 2D layered chalcogenides is the result of their intrinsic high ionic conductivity, high surface area, inherent chemical stability (under a variety of pH conditions) and propensity for charge storage^9^,^10^.

In this work, an electroactive metal oxide BiVO_4_ is combined with few-layered nanostructured (NS) MoS_2_ as a capacitive component to form a hybrid structure. It should, however, be noted that over the last few years several studies that quantify the capacitive storage of MoS_2_ have been reported^11^,^12^,^13^,^14^,^15^,^16^,^17^,^18^,^19^,^20^,^21^,^22^; these typically use MoS_2_ in conjunction with a variety of carbon allotropes, predominantly graphene (r-GO). Interestingly, as yet MoS_2_ has not been coupled with a viable electrochemical component to form a hybrid supercapacitor. Table 1 shows a summary of a few different approaches; the specific capacitance values vary between 100 and 550 Fg^−1^ with an average of *ca*. 250 Fg^−1^. Moreover this value is highly dependent on type of MoS_2_ (bulk *vs*. nanostructured), manner of synthesis of nanostructured MoS_2_ (exfoliated *vs*. synthesized from precursors), current density employed and the amount of loading of MoS_2_ to the carbon source.

Another approach to augment the specific capacitance of electric double-layer capacitors is to incorporate a new electroactive material along with a potent capacitive component to enable high energy density supercapacitors while maintaining high power operations. It has been reported that MoS_2_ suffers from low conductivity, low theoretical specific capacity and easy restacking of the sheets^23^,^24^. Despite the aforementioned limitations of MoS_2_, it is being employed in electrochemical devices *albeit* in conjunction only with carbon nanostructures so as to overcome these limitations.

As mentioned above, here we report a BiVO_4_/MoS_2_ composite, with the first ever usage of bare MoS_2_ as the capacitive component. The MoS_2_ has been synthesized from thermolysis of (NH_4_)_2_MoS_4_ in the presence of H_2_. The resulting MoS_2_/BiVO_4_ nanocomposite has demonstrated much larger values of charge storage, longer discharge times and improved cycling stability in comparison to pure BiVO_4_ itself, or graphene/BiVO_4_ composites. NS MoS_2_/BiVO_4_ composites have been found to be superior to graphene/BiVO_4_ composites, and hence a promising candidate for supercapacitor applications.

## Results and Discussion

In this study, three types of samples were investigated: pure BiVO_4_, a composite of 2.5 wt% graphene/BiVO_4_ and a composite of 2.5 wt% NS MoS_2_/BiVO_4_. These are labeled as BiVO_4_, BiVO_4_-G and BiVO_4_-M for succinctness in the following sections. Details of the synthesis of all these samples are discussed in the methods section.

**Figure 1:**
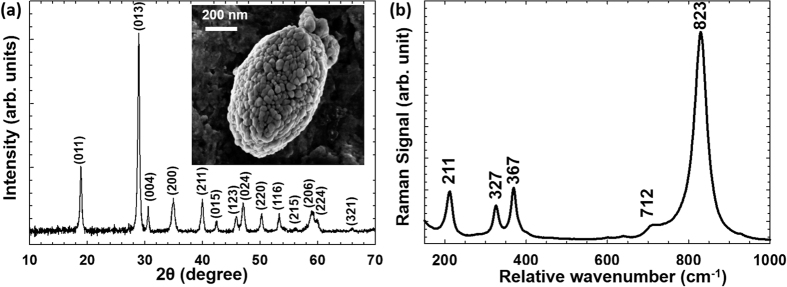
(**a**) XRD pattern of BiVO_4_, in the inset: SEM image of solvothermally synthesized BiVO_4_ and (**b**) Raman spectrum indicating various vibrational modes of BiVO_4_.

BiVO_4_ was synthesized by a solvothermal route. The purity and crystallinity of solvothermally synthesized BiVO_4_ was analyzed by high-resolution X-ray diffraction (XRD), Fig. 1a. The peaks in the diffractogram could be indexed according to JCPDS card no. 75–2480, corresponding to a monoclinic scheelite structure. The inset in Fig. 1a shows an SEM image of BiVO_4_. It can be seen that 10–20 nm diameter spherical BiVO_4_ particles agglomerate to form larger porous oval shaped clusters with average size in the range 200–300 nm. Figure 1b shows a Raman spectrum of BiVO_4_. The most intense Raman band at around 823 cm^−1^ is assigned to the V-O symmetric stretching mode, while the weak shoulder at around 712 cm^−1^ is due to V-O antisymmetric stretch. The band at 211 cm^−1^ is related to external mode (translation/rotation) of BiVO_4_, and the bands at 367 and 327 cm^−1^ are ascribed to the symmetric and asymmetric deformation modes of the VO_4_^3−^ tetrahedron, respectively^25^.

**Figure 2:**
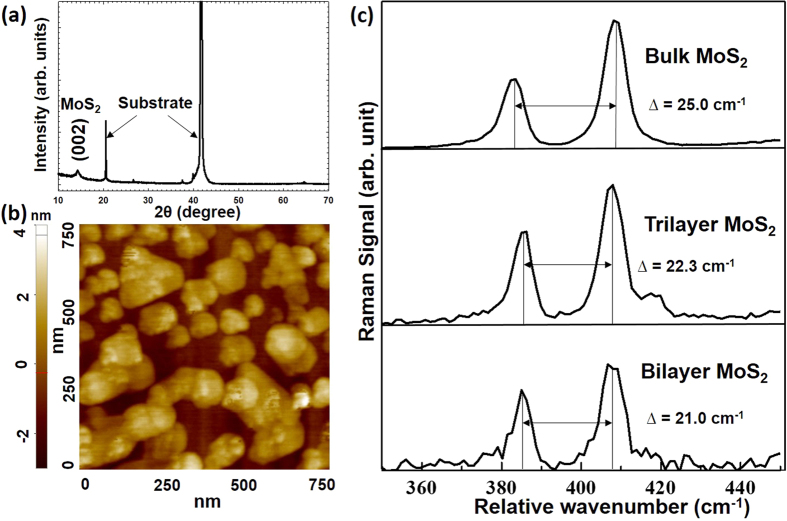
(**a**) XRD showing (002) peak of MoS_2_, (**b**) AFM topographic image of nanostructured MoS_2_ grown on sapphire, and (**c**) Raman spectra of bilayer, trilayer and bulk MoS_2_.

Figure 2a shows the XRD profile of the nanostructured MoS_2_ grown on sapphire via a two-step thermolysis process. The diffraction pattern from the sample ((002) peak) is indexed to MoS_2_, indicating periodicity along the *c-*axis. All other peaks in the XRD correspond to *c-*sapphire (substrate). The samples, after the first annealing step, were subjected to a second annealing at 1000 °C in the mixture of N_2_ and sulfur. The addition of sulfur before the second annealing process greatly improved the crystallinity^26^.

An atomic force microscopy (AFM) image of the MoS_2_/sapphire sample obtained after second annealing in the presence of sulfur is shown in Fig. 2b. From the height of the flakes we can infer the presence of monolayer, bilayer and trilayer regions along with bulk MoS_2_, Fig. S1. The lateral dimension of the flakes are about 50–200 nm.

Figure 2c shows the Raman spectra for MoS_2_ flakes of different thickness on sapphire substrates, obtained using laser excitation at 532 nm. The energy difference (∆) between two Raman peaks (modes A_1g_ and E_2g_) can be used to identify the number of MoS_2_ layers on the substrate^26^. The values of ∆ obtained for different parts of the sample are in the range 21–23 cm^−1^ and 25 cm^−1^, which suggests the presence of nanostructured MoS_2_ (mono/bi/tri-layer) and bulk respectively^26^. It should be noted that normally the spatial resolution of the Raman spectra, being limited by the laser spot size, is poorer than that of an AFM. So the Raman signal is actually averaged over different MoS_2_ flakes even when it is acquired from a single ‘spot’. We have therefore observed that it is difficult to differentiate a monolayer flake from a bilayer flake if the lateral sizes of the flakes are too small using only Raman spectroscopy. As such, further characterization was done by Raman imaging of a larger area (8μm x 8μm) of MoS_2_ on sapphire sample (Supporting Information Fig. S2). The image was acquired using a range of minimum value of 383.6 cm^−1^ (belonging to ideally bulk MoS_2_) to a maximum value of 384.8 cm^−1^ (ideally belonging to few layers of MoS_2_). As such, most of the image depicts that the sample shows a Raman signal closer to that of few layers of MoS_2_. From combined information provided by Raman mapping and AFM, it can therefore be concluded that the MoS_2_ film on the substrate consists of mostly nanostructured MoS_2_ and a few thicker flakes (≥5 monolayers).

**Figure 3:**
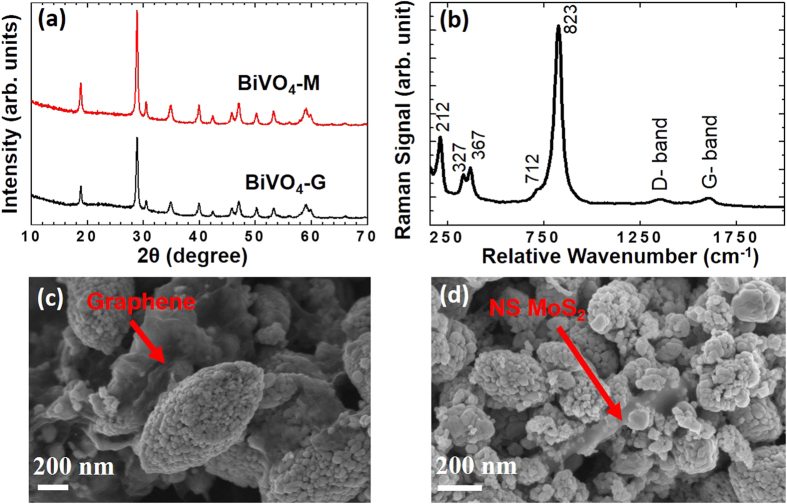
(**a**) XRD profiles of BiVO_4_-G and BiVO_4_-M, all peaks can be indexed to BiVO_4_ which indicates chemical integrity of BiVO_4_ is intact, (**b**) Raman spectrum of BiVO_4_-G showing vibrational features of both BiVO_4_ and graphene, (**c**,**d**) show SEM images of BiVO_4_-G and BiVO_4_-M.

To effectively recover nanostructured MoS_2_ from sapphire, the sample was simply peeled off using a surgical blade. Following this recovery, the MoS_2_/BiVO_4_ composite was prepared via ultrasonication. 2.5wt% NS MoS_2_ was loaded on BiVO_4_. Analogously, a graphene/BiVO_4_ composite was also prepared by ultrasonicating commercially-procured graphene with BiVO_4_. The two components in the composite (the 2D layered material and BiVO_4_) are expected to interact via van der Waals’ interactions. Figure 3a, shows XRD profiles of BiVO_4_-G and BiVO_4_-M, all the peaks can be indexed to BiVO_4_. XRD patterns show well resolved peaks of monoclinic BiVO_4_, and there were no deleterious effects observed due to ultrasonication. The peaks due to diffraction from the MoS_2_ lattice planes were however not observed in the diffractogram, perhaps due to the very small amount of MoS_2_ present and lack of a preferential orientation following delamination from the original sapphire substrate. Figure 3b shows Raman spectrum of BiVO_4_-G obtained using laser excitation at 532 nm. All vibrational modes of BiVO_4_ are observed along with vibrational signatures of graphene (D- and G- bands). Figure 3c and d show SEM images of BiVO_4_-G and BiVO_4_-M composites respectively. These images clearly show the two separate phases of capacitive components and BiVO_4_. The energy-dispersive X-ray pattern for BiVO_4_-M confirmed the presence of Bi, V and Mo (Fig. S3).

### Electrochemical characterization

The electrochemical behavior of all three samples (BiVO_4_, BiVO_4_-M and BiVO_4_-G) was evaluated by performing cyclic voltammetry, charge discharge and cycling stability, using the same working electrode.

**Figure 4:**
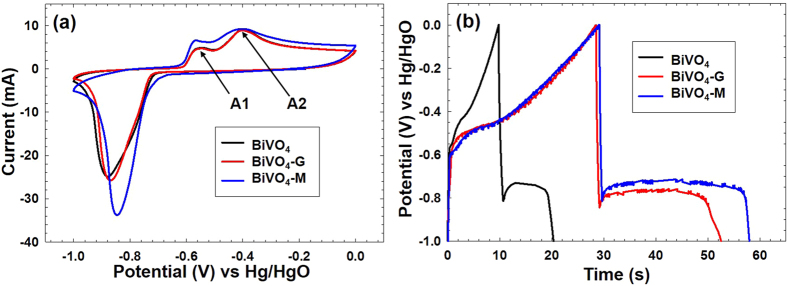
(**a**) CV curves of BiVO_4_, BiVO_4_-G and BiVO_4_-M at a scan rate of 20 mVs^−1^, (**b**) CD profile of BiVO_4_, BiVO_4_-G and BiVO_4_-M at a current density of 7 Ag^−1^.

Cyclic voltammetry measurements were performed at various scan rates from 5 to 50 mVs^−1^, the data presented in Fig. 4a is for 20 mVs^−1^ scans (the other scans are shown in the supporting information). Redox peaks in the CV curve can be attributed to quasi-reversible faradaic process (Bi^3+^ ↔ Bi^0^). For bare BiVO_4_, a single peak with high current was assigned to reduction of Bi^3+^ to Bi^0^ (−0.85 V), on the other side, two anodic peaks were obtained at -0.52 V and -0.4 V for the oxidation^27^,^28^. The oxidation peak A1 is assigned to the oxidation of Bi^0^ to Bi^+^ and peak A2 is assigned to the oxidation of Bi^+^ to Bi^3+^. Further, the CV profiles for BiVO_4_-G and BiVO_4_-M in Fig. 4a matched well with CV curve of pristine BiVO_4_ indicating redox peaks were due to BiVO_4_ and the presence of MoS_2_ or graphene did not affect its electrochemical response. No extra peaks are visible for BiVO_4_-M suggesting that the small fraction of MoS_2_ did not show any charge transfer activity in the applied potential window that was operative. With increasing scan rates the oxidation peak potentials shift towards positive direction and reduction peak potentials shift towards negative direction, which is mainly attributed to IR drop component, which dominates at higher current values. The kinetics of interfacial faradaic redox reaction was rapid enough, as an increase in current response at higher scan rates was observed, (see Fig. S4a in the supporting information)^28^.

**Figure 5:**
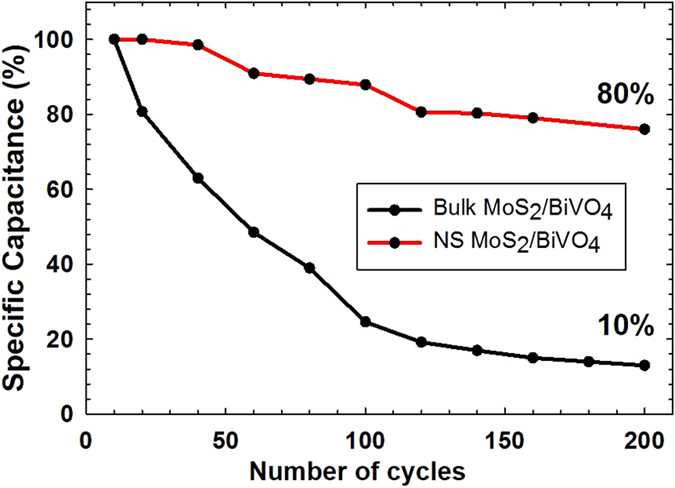
Cycling performance in terms of specific capacitance (%) of Bulk MoS_2_/BiVO_4_ and BiVO_4_-M at a current density 3 Ag^−1^.

The electrochemical capacitance of BiVO_4_, BiVO_4_-M and BiVO_4_-G was evaluated by galvanostatic charge discharge (CD) measurements. For all the samples, 5 cycles for all current densities were carried out; Fig. 4b presents the data of the 2^nd^ cycle in each case. CD profile of BiVO_4_ indicated pseudo-capacitive nature of material for charge storage applications. Similar to BiVO_4,_ CD curves of BiVO_4_-M and BiVO_4_-G (Fig. 4b) between potential window −1.0 V to 0.0 V appeared to be non-symmetric, insinuating battery behavior and showing the IR drop. Despite the steep voltage drop, prolonged plateau of voltage output was observed, which is due to the involvement of faradaic process in BiVO_4._ Using equation, 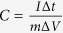 specific capacitance values were obtained. Specific capacitance of BiVO_4_-M was found to be (212 Fg^−1^ at 3 Ag^−1^) which is not only higher than bare BiVO_4_ (108 Fg^−1^ at 3 Ag^−1^) but at higher current densities it is surpassing specific capacitance obtained for BiVO_4_-G (Table S1). Figure 4b shows CD profiles of pristine BiVO_4_, BiVO_4_-M, and BiVO_4_-G at a current density 7 Ag^−1^, which clearly indicates nanostructured MoS_2_ composite with BiVO_4_ has maximum specific capacitance. Generally, the boost of current density results in fading of specific capacitance which is primarily due to inaccessibility of inner electroactive sites to the electrolyte ions due to diffusion limitations (Fig. S4b)^29^. However, even at higher current densities the specific capacitance values are found to be 166.6 Fg^−1^ and 156 Fg^−1^ at 10 Ag^−1^ and 15 Ag^−1^, respectively, which are impressive. High capacitance values at faster charging rates can be explained by the fact that transition metal dichalcogenides show higher ionic diffusivity as a consequence of large anionic polarizability^30^. For comparison, 2.5 wt% bulk MoS_2_ loaded BiVO_4_ was also prepared via ultrasonication and electrochemical behavior of the same was studied. Although the capacitance values of bulk MoS_2_/BiVO_4_ were higher than nanostructured MoS_2_/BiVO_4_, the composite with the bulk appears to show poor cycling stability (Fig. 5). It was observed that discharge capacity was retained only 10% up to 200 cycles for BiVO_4_-Bulk MoS_2_ composite. On the other hand, BiVO_4_-M exhibited better long term stability, as it could retain ~80% of the initial value after 200 cycles, for the same current density used for charging (3 Ag^−1^). The excellent structural and mechanical stability shown by BiVO_4_-M composite can be ascribed to the high elasticity of nanostructured MoS_2_. As depicted by the AFM topographic image, the synthesis method reported here yielded MoS_2_ with a lateral particle size of a few 100 nm. It has been purported that as the lateral size of MoS_2_ is decreased, there is a higher preponderance of step-edges, and low-coordination edge and corner atoms as compared to basal plane atoms^31^. The effect of these states dominate over those of the basal atoms and can contribute to higher charge storage sites. In addition, since the particles are made up of a few-layered sheets, it has been highlighted by Chhowalla *et al.*^9^ that such loosely stacked sheets are able to accommodate structural changes in a better manner upon cycling when compared to bulk MoS_2._ The latter has shown large structural instability especially as anodes in lithium ion batteries. As such, when evaluated in unison, the explicitly synthesized small lateral-dimensioned particles which consist of only a few layers of MoS_2_ appear to contribute substantially to the charge storage, discharge times and cycling stability of the MoS_2_/BiVO_4_ composite synthesized in this report.

## Methods

Analytical reagent grade ammonium metavanadate (NH_4_VO_3_), sodium dodecyl sulfate (SDS) and anhydrous glycerol were purchased from Merck, India. Polyvinlydene fluoride (PVDF), N-methyl-2-pyrrolidinone (NMP) and activated carbon (AC) were purchased from Himedia, India whereas bismuth nitrate pentahydrate (Bi(NO_3_)_3_.5H_2_O) was purchased from Sigma Aldrich. Research grade graphene dispersion in water was procured from US Research Nanomaterials Inc. All chemicals were used as received without further purification.

### Synthesis of monoclinic-BiVO_4_

BiVO_4_ was prepared by following earlier work reported by Khan *et al.*^28^ Typically, 3 mmol of SDS was dissolved in 40 mL of solvent (DI water and glycerol with volume ratio 1:1) in a flask at room temperature. 1 mmol Bi(NO_3_)_3_.5H_2_O and 1 mmol NH_4_VO_3_ was added to the above clear solution under constant stirring in sequence. After stirring for 7 minutes, the solution was transferred into a stainless steel autoclave with a Teflon liner and heated at 160 °C for 18 hours. After cooling to room temperature, the reaction mixture was centrifuged and the pellet was washed with water and ethanol. Finally, the product was dried under vacuum at 70 °C for *ca*. 5 hours.

### Synthesis of nanostructured MoS_2_

Synthesis of few layered MoS_2_ was successfully achieved by following a published procedure with major modifications^26^. 25 mg of (NH_4_)_2_MoS_4_ was dissolved in 20 mL DMF and sonicated for 20 minutes. 10 μL of this precursor solution was spin coated on sapphire at 3000 rpm for 60 seconds. The substrate was then heated on a hot plate at 120 °C for 30 minutes. The annealing process was performed in a homemade rapid thermal annealing furnace. The freshly prepared thin (NH_4_)_2_MoS_4_ layer was placed on the graphite sample holder in the tube furnace flowing with a gas mixture N_2_/H_2_. It was then heated at 500 °C and was maintained for 60 minutes under constant flow of N_2_/H_2_ to efficiently remove residual solvent, ammonia molecules, and other byproducts from the precursor. After this step, the furnace was cooled down to room temperature, following which additional sulfur was introduced to the sample holder and the gas environment was changed to N_2_ and after 20 minutes purging to remove any air introduced, the temperature was raised to 1000 °C and was maintained for 30 minutes.

### Synthesis of 2.5 wt% nanostructured MoS_2_/BiVO_4_ composite

0.2 mg of nanostructured MoS_2_ was recovered via scraping with a surgical blade and was dispersed in 15 mL water. To this 8 mg BiVO_4_ was added, and the dispersion was sonicated for 8 hours. The composite was recovered after centrifugation and dried under vacuum at 70 °C for 5 hours.

### Synthesis of 2.5 wt% graphene/BiVO_4_ composite

1 mg of graphene and 40 mg of BiVO_4_ were dispersed in 30 mL ethanol, and the dispersion sonicated for 8 hours. The composite was recovered by evaporating the solvent on a hot plate at 90 °C.

### Characterization

Powder X-ray diffraction (XRD) measurements from 10° to 70° 2θ were recorded using a PANalytical X’pertpro diffractometer with monochromatic Cu Kα source (λ = 1.54056 Å) operating at 40 kV and 30 mA. The elemental composition and surface morphologies of the samples were investigated by Field Emission Scanning Electron Microscopy (FE-SEM) on a Zeiss Ultra FEG 55 instrument at 5 kV operating voltage. Cyclic voltammetry and galvanostatic charge-discharge (CD) studies of the composites were carried out using Bio-Logic VMP3 Galvanostat/Potentiostat Instruments at room temperature.

### Electrode material and electrochemical tests

Electrochemical measurements were carried out in a 2 M aqueous NaOH in a three electrode cell at room temperature. Hg/HgO/Ca(OH)_2_ and platinum plate were used as reference and counter electrodes, respectively. The active electrode was prepared by mixing electroactive material (80 wt%), activated carbon (15 wt%) and polyvinylidene fluoride (5 wt%) with 1 mL of NMP to form a slurry which was coated and dried on a small piece of graphite plate (area of coating, 1 cm^2^).

## Additional Information

**How to cite this article**: Arora, Y. *et al.* Nanostructured MoS_2_/BiVO_4_ Composites for Energy Storage Applications. *Sci. Rep.* **6**, 36294; doi: 10.1038/srep36294 (2016).

**Publisher’s note:** Springer Nature remains neutral with regard to jurisdictional claims in published maps and institutional affiliations.

## Supplementary Material

Supplementary Information

## Figures and Tables

**Table 1:** Comparison of supercapacitor performance of MoS_2_/carbon nanostructures and bare MoS_2_.

Type of MoS_2_	Type of carbon nanostructure	Loading %	Electrolyte	Current density/Scan rate	Specific capacitance
Bulk MoS_2_^11^	Porous carbon tubes	Not provided	3.0 M KOH	1 Ag^−1^	**210** **Fg**^**−1**^
Few layered MoS_2_^12^	Graphene	Not provided	1 M HClO_4_	1 Ag^−1^	**243** **Fg**^**−1**^
Few layered MoS_2_^13^	Graphene (r-GO)	17.6 wt% MoS_2_ loaded on r-GO	1 M HClO_4_	10 mVs^−1^	**265** **Fg**^**−1**^
Few layered MoS_2_^14^	Graphene	Not provided	1 M HClO_4_	20 mVs^−1^	**282** **Fg**^**−1**^
MoS_2_ (bulk)^15^	Graphene (r-GO)	50% r-GO loading	1 M H_2_SO_4_	5 mVs^−1^	**416** **Fg**^**−1**^
3-D tubular MoS_2_^16^	Polyaniline	PANI is 60% loaded	1 M H_2_SO_4_	0.5 Ag^−1^	**552** **Fg**^**−1**^
MoS_2_ nanosheets (bulk)^17^	—	—	1 M Na_2_SO_4_	0.5 Ag^−1^	**92** **Fg**^**−1**^
MoS_2_ nanospheres (bulk)^18^	—	—	1 M KCl	1 Ag^−1^	**122** **Fg**^**−1**^
MoS_2_ nanosheets^19^	—	—	1 M Na_2_SO_4_	1 Ag^−1^	**129.2** **Fg**^**−1**^
Monolayer MoS_2_^20^	—	—	0.5 M H_2_SO_4_	20 mVs^−1^	**~140** **Fg**^**−1**^
MoS_2_ nanoflowers^21^	—	—	1 M KCl	1 Ag^−1^	**168** **Fg**^**−1**^
Mesoporous MoS_2_^22^	—	—	1 M KCl	1 mVs^−1^	**403** **Fg**^**−1**^
*Few layered nanostructured MoS*_*2*_*/BiVO*_*4*_ *– This work*	*—*	***2.5 wt% MoS***_***2***_***loaded on BiVO***_***4***_	***2 M NaOH***	***1*** ***Ag***^***−1***^	***610*** ***Fg***^***−1***^
