# Supplementary material for: Nanostructured MoS2/BiVO4 Composites for Energy Storage Applications
Source: Sci Rep. 2016 Nov 3;6:36294. doi: 10.1038/srep36294 (PMC5093765; doi:10.1038/srep36294)
Supplement: Supplementary Information [file srep36294-s1.doc]

**Supporting Information**

**Nanostructured MoS_2_/BiVO_4_ Composites for Energy Storage Applications**

Yukti Arora^a^, Amit P Shah^b^, Shateesh Battu^c^, Carina B Maliakkal^b^, Santosh Haram^c*^, Arnab Bhattacharya^b*^, and Deepa Khushalani^a*^

^a^Department of Chemical Sciences, Tata Institute of Fundamental Research, Mumbai-400005, India

^b^Department of Condensed Matter Physics and Materials Science, Tata Institute of Fundamental Research, Mumbai-400005, India

^c^Department of Chemistry, University of Pune, Pune-411007, India

*email: [khushalani@tifr.res.in](mailto:khushalani@tifr.res.in); [arnab@tifr.res.in](mailto:arnab@tifr.res.in); [santoshharam@gmail.com](mailto:santoshharam@gmail.com)

**Figure S1**. AFM image and height line profile of nanostructured MoS_2_ grown on sapphire (substrate) to show there are regions of mono/bi/tri layers as well as bulk, N corresponds to number of MoS_2_ mono layers.

**Figure S2.** Raman Image of MoS_2_/sapphire sample obtained after second annealing where the color indicates the center of the E_2g_ peak. The lighter region has the E_2g_ peak centered at ~384.8 cm^-1^ corresponding to nanostructured (NS) MoS_2_ (In these areas the delta is in the range 21-23 cm^-1^). In the darker region of the image the center of E_2g_ peak is ~383.6 cm^-1^ (and delta is ≥25 cm^-1^) indicating bulk MoS_2_.

**Figure S3**. EDX spectra showing presence of Bi, V and S.

**Figure S4.** (**a**) CV of BiVO_4_-M at higher scan rates and (**b**) CD profiles of BiVO_4_-M at higher current densities.

**Table S1.** Specific capacitance values of BiVO_4_-G and BiVO_4_-M at various current densities.
